# Inhibition Mechanism of the Intracellular Transporter Ca^2+^-Pump from Sarco-Endoplasmic Reticulum by the Antitumor Agent Dimethyl-Celecoxib

**DOI:** 10.1371/journal.pone.0102083

**Published:** 2014-07-08

**Authors:** Ramón Coca, Fernando Soler, Ernesto Cortés-Castell, Vicente Gil-Guillén, Francisco Fernández-Belda

**Affiliations:** 1 Departamento de Medicina Clínica, Universidad Miguel Hernández en Campus de San Juan, Alicante, Spain; 2 Departamento de Bioquímica y Biología Molecular A, Universidad de Murcia en Campus de Espinardo, Murcia, Spain; 3 Departamento de Farmacología, Pediatría y Química Orgánica, Universidad Miguel Hernández en Campus de San Juan, Alicante, Spain; Purdue University, United States of America

## Abstract

Dimethyl-celecoxib is a celecoxib analog that lacks the capacity as cyclo-oxygenase-2 inhibitor and therefore the life-threatening effects but retains the antineoplastic properties. The action mechanism at the molecular level is unclear. Our *in vitro* assays using a sarcoplasmic reticulum preparation from rabbit skeletal muscle demonstrate that dimethyl-celecoxib inhibits Ca^2+^-ATPase activity and ATP-dependent Ca^2+^ transport in a concentration-dependent manner. Celecoxib was a more potent inhibitor of Ca^2+^-ATPase activity than dimethyl-celecoxib, as deduced from the half-maximum effect but dimethyl-celecoxib exhibited higher inhibition potency when Ca^2+^ transport was evaluated. Since Ca^2+^ transport was more sensitive to inhibition than Ca^2+^-ATPase activity the drugs under study caused Ca^2+^/P_i_ uncoupling. Dimethyl-celecoxib provoked greater uncoupling and the effect was dependent on drug concentration but independent of Ca^2+^-pump functioning. Dimethyl-celecoxib prevented Ca^2+^ binding by stabilizing the inactive Ca^2+^-free conformation of the pump. The effect on the kinetics of phosphoenzyme accumulation and the dependence of the phosphoenzyme level on dimethyl-celecoxib concentration were independent of whether or not the Ca^2+^–pump was exposed to the drug in the presence of Ca^2+^ before phosphorylation. This provided evidence of non-preferential interaction with the Ca^2+^-free conformation. Likewise, the decreased phosphoenzyme level in the presence of dimethyl-celecoxib that was partially relieved by increasing Ca^2+^ was consistent with the mentioned effect on Ca^2+^ binding. The kinetics of phosphoenzyme decomposition under turnover conditions was not altered by dimethyl-celecoxib. The dual effect of the drug involves Ca^2+^-pump inhibition and membrane permeabilization activity. The reported data can explain the cytotoxic and anti-proliferative effects that have been attributed to the celecoxib analog. Ligand docking simulation predicts interaction of celecoxib and dimethyl-celecoxib with the intracellular Ca^2+^ transporter at the inhibition site of hydroquinones.

## Introduction

Cyclo-oxygenase-2 (COX)-2 is the inducible isoform of prostaglandin G/H-synthase [EC1.14.99.1], the bifunctional enzyme involved in the transformation of arachidonic acid into prostaglandin H_2_ [1]. It is selectively expressed in certain tissues and also induced during inflammation. In this regard, celecoxib (CLX) is a non-steroidal anti-inflamatory drug with specificity as COX-2 inhibitor that was developed to avoid the gastrointestinal side effects induced by non-selective inhibitors [2].

Apart from anti-inflammatory and analgesic properties, it was found that chemically induced carcinogenesis in rat was inhibited by CLX in the diet [3], [4]. These early experiments pointed to the possibility of a beneficial effect in cancer prevention and treatment. Indeed, COX-2 upregulation and/or abnormal expression have been reported in several types of cancer [5]–[8] and elevated COX-2 expression in tumors is associated with increased angiogenesis, tumor invasion and resistance to apoptosis. Clinical studies also supported the anticancer activity of CLX [9], [10], although some cardiovascular and thrombotic events have been described [11], [12]. The unwanted effects have been attributed to selective COX-2 inhibition in the blood vessels, leading to vasoconstriction and concomitant platelet aggregation mediated by the COX-1 activity [13].

Studies on rat colon carcinogenesis showed that CLX in the diet, equivalent to 3.5 µg/ml in blood serum (∼9 µM CLX) was needed to provoke an antitumor effect, whereas the anti-inflammatory dose was 0.8 µM [3]. This was consistent with clinical data showing that 800 mg CLX per day was necessary to reduce the number of colorectal polyps, in contrast to the recommended anti-inflammatory dose of 100 to 200 mg [9]. Moreover, assays with different tumor cell lines showed that the cytotoxic effect of CLX required >20 µM concentrations although the same effect was observed in COX-deficient fibroblasts [14]. It is now accepted that moderate micromolar concentrations are needed to exert anti-proliferative action and therefore the cytotoxic effect induced by micromolar CLX is not related with COX-2 inhibition [15].

The irreversible rise of cytosolic Ca^2+^ when PC-3 cells were exposed to CLX revealed a link between CLX and cytotoxicity. The effect was attributed to inhibition of the sarco-endoplasmic reticulum Ca^2+^-ATPase (SERCA) [16]. In fact, CLX and also dimethyl-celecoxib (DMC) induced Ca^2+^ discharge from intracellular stores in a glioblastoma cell line as occurred under the presence of the high affinity SERCA inhibitor thapsigargin (TG) [17]. When the COX-2 inhibition capacity and the apoptotic activity of several CLX derivatives were analyzed it was disclosed that both functions operate separately [18]. Thus, DMC displayed reduced ability as COX-2 inhibitor but high potency as apoptotic inducer [15], [18].

It has been shown that DMC, and in a lesser extent CLX, put in motion the endoplasmic reticulum stress response mediated by sustained elevation of cytosolic free Ca^2+^ leading to cell death by apoptosis. The process was observed in several tumor cell lines including glioblastoma, breast carcinoma, pancreatic carcinoma, Burkitt's lymphoma and multiple myeloma [17]. Further evidence comes from xenografted tumor cells in animal model showing anti-proliferative effect of CLX and DMC [17], [19].

SERCA, also termed Ca^2+^-pump from sarco-endoplasmic reticulum, is the main intracellular transporter of most eukaryotic cells involved in the removal of cytosolic Ca^2+^. It builds up and maintains a >10^4^-fold concentration gradient owing to the uphill transport of Ca^2+^ from the cytosol to the luminal space of the sarcoplasmic reticulum (SR) [20]. Refilling of the intracellular organelle is instrumental in the generation of cytosolic Ca^2+^ signals that give rise to a wide variety of cellular responses [21].

Since SERCA inhibition unleashes apoptotic cell death, the aim of the present study was to shed light on the effect of DMC as antitumor agent on SERCA catalytic and transport functions. Information on the interaction site of CLX and DMC was also pursued.

## Materials and Methods

This study was performed in accordance with the European Union Council Directive of 22 September 2010 (2010/63/EU) and reviewed and approved by the Ethical Committee of the University of Murcia.

### Chemicals and other materials

DMC was obtained from Sigma-Aldrich (Madrid, Spain) and CLX was from BioVision (Milpitas, CA USA). *Streptomyces chartreusensis* A23187 from Calbiochem and the Ca^2+^ standard solution Titrisol were provided by Merck KGaA (Madrid, Spain). ^45^CaCl_2_, [γ-^32^P]ATP and [6-^3^H(N)]D-glucose were New England Nuclear products from PerkinElmer (Madrid, Spain). The liquid scintillation cocktail Optiphase HiSafe 3 was also from PerkinElmer. HAWP type nitrocellulose filters with 0.45-µm pore size for vacuum filtration were from Merck Millipore (Madrid, Spain). All other analytical grade reagents were obtained from Sigma-Aldrich.

### Ca^2+^ in the media

Defined free Ca^2+^ concentrations were adjusted by adding appropriate volumes of CaCl_2_ and EGTA stock solutions as previously described [22]. The computer program for calculation took into account the absolute stability constant for the Ca^2+^-EGTA complex [23], the EGTA protonation equilibria [24], the presence of Ca^2+^ ligands and the pH in the medium. A nominally Ca^2+^-free medium was established by including excess EGTA without any Ca^2+^ addition. Free Ca^2+^ concentration was expressed as negative logarithm of free Ca^2+^ (pCa). For instance, 1 mM EGTA and 0.686 mM CaCl_2_ give a pCa value of 6.0.

### Microsomal preparation and protein content

The starting material was hind leg skeletal muscle from adult female New Zealand rabbit. Right-side vesicles mainly derived from longitudinal tubules of the SR membrane were obtained as described by Eletr and Inesi [25]. Isolated vesicles were aliquoted and stored at −80°C for further use. Samples of SR vesicles as prepared (native vesicles) or in the presence of Ca^2+^ ionophore A23187 (leaky vesicles) were used. SERCA1a is by far the most abundant protein in the microsomal preparation from rabbit skeletal muscle [26] and the only one bearing high affinity Ca^2+^ sites. The experimental protocols described in this study, i.e., rates of active Ca^2+^ transport and Ca^2+^-dependent ATP hydrolysis, Ca^2+^ binding to the high affinity transport sites and phosphoenzyme (EP) formed from ATP in the presence of Ca^2+^ have been successfully applied by others for the functional characterization of SERCA using the abovementioned microsomal fraction [27]. The concentration of SR membrane refers to mg of total protein per ml and was measured by the Lowry et al. procedure [28]. Bovine serum albumin was used as a standard protein.

### Active Ca^2+^ transport

The initial rate of Ca^2+^ accumulation inside native vesicles was measured at 25°C by a radioactive procedure [29]. The initial experimental medium consisted of 20 mM 4-morpholinepropanesulfonic acid (Mops), pH 7.0, 80 mM KCl, 5 mM MgCl_2_, 1 mM EGTA, 0.686 mM [^45^Ca^2+^]CaCl_2_ at ∼2 000 cpm/nmol, 0.02 mg/ml native vesicles and 5 mM potassium oxalate in the absence or presence of a given drug concentration. The transport process was started by adding 1 mM ATP and stopped at different times by filtering under vacuum 0.5 ml aliquots. Thereafter, the filters were rinsed with 5 ml of ice-cold medium containing 20 mM Mops, pH 7.0 and 1 mM LaCl_3_ and the retained radioactivity was evaluated by liquid scintillation technique.

### ATP hydrolysis

The initial rate of inorganic phosphate (P_i_) release was measured at 25°C with a malachite green reagent [30]. The standard reaction mixture for measurements with native vesicles was 20 mM Mops, pH 7.0, 80 mM KCl, 5 mM MgCl_2_, 1 mM EGTA, 0.686 mM CaCl_2_, 0.05 mg/ml native vesicles, 5 mM potassium oxalate and 1 mM ATP in the absence or presence of a certain drug concentration. Aliquots of the reaction medium containing 0.02 ml were withdrawn at different time intervals and mixed with 0.8 ml of malachite green reagent to allow color development. Measurements with leaky vesicles were performed in the reaction medium described above but including 4 µM A23187 instead of potassium oxalate. The Ca^2+^–dependent activity was calculated by subtracting the hydrolytic activity measured in the absence of Ca^2+^.

### Ca^2+^ binding

Equilibrium experiments of Ca^2+^ binding in the absence of ATP were performed by sample filtration using a double-labeling radioactive procedure to discount unspecific Ca^2+^ [31]. The incubation medium contained 20 mM Mops, pH 7.0, 80 mM KCl, 3 mM MgCl_2_, 0.1 mM EGTA, 1 mM glucose, 0.2 mg/ml native vesicles and 50 µM DMC when indicated. After equilibration at 25°C for 5 min, aliquots of 0.6 ml were supplemented with a certain volume of medium containing 20 mM Mops, pH 7.0, 80 mM KCl, 3 mM MgCl_2_, 0.1 mM EGTA, 3 mM [^45^Ca]CaCl_2_ at ∼10 000 cpm/nmol and 1 mM [^3^H]glucose at ∼30 000 cpm/nmol. Final pCa ranged from 7.0 to 5.0. Samples were incubated at 25°C for 3 min and then 0.5 ml aliquots (0.1 mg protein) were filtered under vacuum without any rinsing. Radioactive tracers ^45^Ca^2+^ and ^3^H in the filters were measured by liquid scintillation counting.

### Drug effect on Ca^2+^-loaded vesicles

Native vesicles were actively loaded at 25°C with radioactive Ca^2+^ in a medium containing 20 mM Mops, pH 7.0, 80 mM KCl, 5 mM MgCl_2_, 0.2 mM EGTA, 0.138 mM [^45^Ca]CaCl_2_ at ∼10 000 cpm/nmol, 0.05 mg/ml native vesicles and 1 mM ATP. When indicated, 50 µM drug, 5 mM EGTA (final pCa 7.9) or 5 mM EGTA plus 50 µM drug was added to the loading medium after 2 min. Then, aliquots of 0.5 ml containing 0.025 mg protein were filtered at different times and the filters were rinsed with 5 ml of cold medium composed of 20 mM Mops, pH 7.0 and 1 mM CaCl_2_.

### EP measurements

Radioactive EP formed from [γ-^32^P]ATP in the presence of Ca^2+^ was measured by the acid quenching technique described by Inesi et al. [27]. All solutions were pre-cooled and the experiments were performed at ice-water temperature. Quenched samples were filtered through nitrocellulose filters and rinsed 5 times with 5 ml each time of quenching solution containing 125 mM perchloric acid and 2 mM sodium phosphate. Radioactive ^32^P retained by the filters was evaluated by liquid scintillation counting.

#### Leaky vesicles in the presence of Ca2+

The initial reaction medium containing 20 mM Mops, pH 7.0, 80 mM KCl, 5 mM MgCl_2_, 1 mM EGTA, CaCl_2_ to give a defined pCa, 0.2 mg/ml SR vesicles, 8 µM A23187 and a given DMC concentration when indicated was distributed in aliquots of 0.5 ml. The phosphorylation reaction was initiated by mixing under vortexing each aliquot with 20 µl of 1 mM [γ-^32^P]ATP at ∼50 000 cpm/nmol to give a final ATP concentration of 40 µM. The phosphorylation reaction was stopped at different times by adding 5 ml of quenching solution.

#### Leaky vesicles in the absence of Ca2+

The initial reaction medium containing 20 mM Mops, pH 7.0, 80 mM KCl, 5 mM MgCl_2_, 1 mM EGTA, 0.2 mg/ml SR vesicles, 8 µM A23187 and a certain DMC concentration when indicated was distributed in 0.5 ml aliquots. The phosphorylation reaction was started by adding 20 µl of 1 mM [γ-^32^P]ATP at ∼50 000 cpm/nmol and 17.2 mM CaCl_2_. Final concentrations after mixing were 40 µM ATP and 0.686 mM CaCl_2_. The phosphorylation reaction was arrested at different time intervals by adding 5 ml of quenching solution.

#### EP decomposition under turnover conditions

The initial phosphorylation medium was 20 mM Mops, pH 7.0, 80 mM KCl, 5 mM MgCl_2_, 1 mM EGTA, 0.686 mM CaCl_2_, 0.4 mg/ml SR vesicles, 16 µM A23187 in the absence or presence of 50 µM DMC. Aliquots of 0.25 ml were mixed with 10 µl of 1 mM [γ-^32^P]ATP at ∼50 000 cpm/nmol. After 5 s, the reaction mixture was 10-fold diluted with 20 mM Mops, pH 7.0, 80 mM KCl, 5 mM MgCl_2_, 1 mM EGTA, 0.686 mM CaCl_2_ and 40 µM ATP and the reaction was halted at serial times by adding 2 ml of 438 mM perchloric acid and 7 mM sodium phosphate. Quenched samples were processed as described above.

### Docking methodology

For protein and ligands processing, the coordinates of the three-dimensional SERCA structure in the absence of Ca^2+^ was obtained from Protein Data Bank (PDB) ID code 2AGV (http://www.rcsb.org). All non-protein components were deleted from the protein structure and the CLX file in pdb format was obtained from PDB ID code 1OQ5. Avogadro software (http://avogadro.openmolecules.net/) [32] was used to build the DMC molecule and optimize the ligands structure. Pdb files were imported into the free graphical user interface AutoDockTools (http://autodock.scripps.edu/resources/adt) [33] for further manipulation. Rotable bonds were set up in the ligands whereas hydrogen atoms were added and partial atomic charges were assigned in ligands and protein. Grid center point was determined from the centroid of BHQ as appears in PDB ID code 2AVG. To obtain the centroid, the Cartesian coordinates for each atom in the ligand were extracted and the average for each dimension was taken. The grid size for docking simulations was set at 25 Å. Docking was carried out with the AutoDock Vina program (http://vina.scripps.edu/) [34]. The results of docking simulations were analyzed with AutoDockTools and molecular graphics images for publication were performed with UCSF Chimera package (http://www.cgl.ucsf.edu/chimera) [35]. The docking methodology was validated with the crystal structure of SERCA co-crystallized with 2,5-di(*tert*-butyl)-hydroquinone (BHQ) and TG (retrieved from PDB ID code 2AGV) by comparing the initial binding conformation of BHQ in the crystal structure and the docked pose obtained from docking simulations.

### Data presentation

Experimental results correspond to mean values of at least three independent determinations, each performed in duplicate and standard deviations are indicated by error bars. Curve fitting was performed by non-linear regression using version 11.0 of the SigmaPlot program from Systat Software (Chicago, IL USA).

## Results

### Turnover parameters

**Figure 1:**
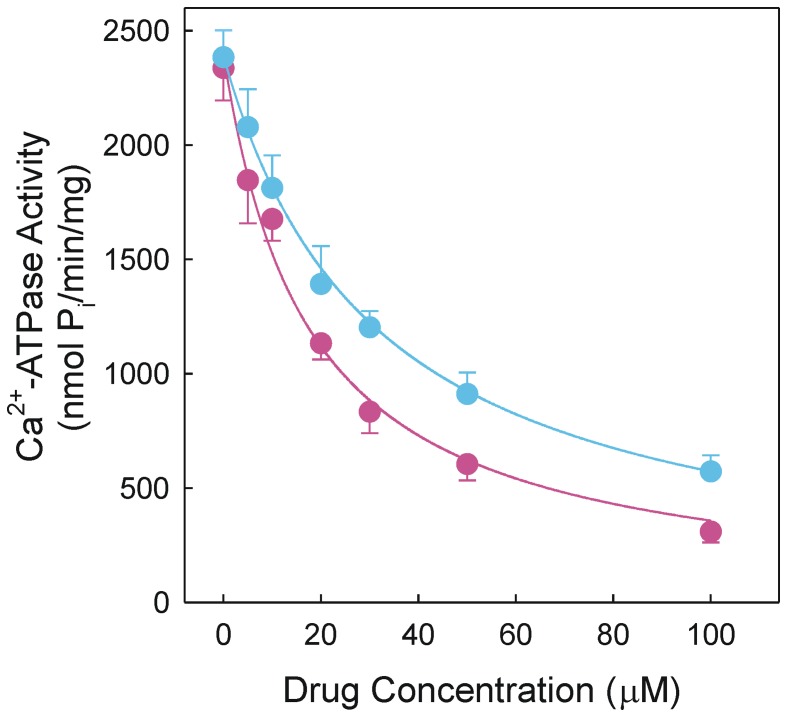
Depedence of Ca^2+^ –ATPase activity on drug concentration in leaky vesicles. The catalytic activity was measured at 25°C in a reaction medium containing 20 mM Mops, pH 7.0, 80 mM KCl, 5 mM MgCl_2_, 1 mM EGTA, 0.686 mM CaCl_2_, 4 µM A23187, 0.05 mg/ml SR vesicles and 1 mM ATP. A given concentration of DMC (blue circles) or CLX (red circles) was included when indicated. The rate of P_i_ release was measured by a discontinuous colorimetric method.

The functional properties of the SR Ca^2+^-pump were initially studied by measuring Ca^2+^-ATPase activity. To this end, SR vesicles leaky to Ca^2+^ were exposed to DMC before ATP was added to initiate the catalytic turnover. The initial rate of the Ca^2+^–dependent ATP hydrolysis was gradually inhibited as the drug concentration was raised and a half-maximum effect was observed at 32 µM (Figure 1). When the parent compound CLX was used, the inhibition profile was similar but the half-maximum inhibition value was 18 µM. The presence of A23187 prevented inhibition of the Ca^2+^-ATPase activity that occurs when free Ca^2+^ is accumulated inside the vesicles.

**Figure 2:**
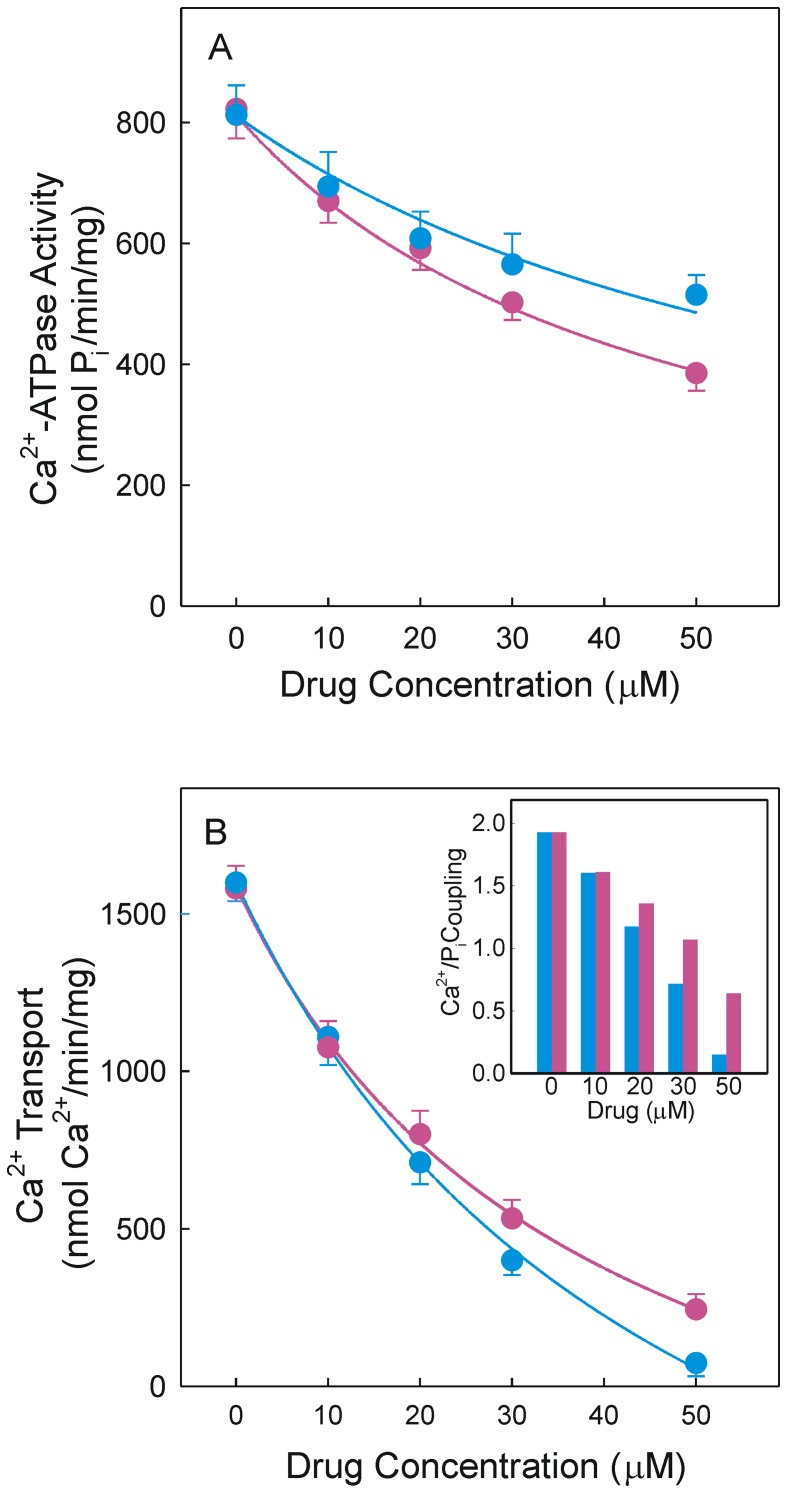
Drug effect on Ca^2+^-ATPase activity, active Ca^2+^ transport and Ca^2+^/P_i_ coupling. Reaction mixtures included native SR vesicles and 5^2+^-ATPase activity (A) or Ca^2+^ transport (B) on DMC (blue circles) or CLX (red circles). Inset, coupling ratios in the presence of DMC (blue bars) or CLX (red bars).

Initial rates of Ca^2+^-ATPase activity and ATP-dependent Ca^2+^ transport were then measured using native vesicles and the presence of the Ca^2+^-trapping oxalate to obtain linear rates of hydrolysis and transport. Here again, Ca^2+^-ATPase inhibition was dependent on drug concentration and was more potent in the presence of CLX (Figure 2A). As a reference, the inhibition was about 53% in the presence of 50 µM CLX and 41% in the presence of 50 µM DMC. Leaky vesicles were more sensitive to inhibition than native vesicles. When the initial rates of Ca^2+^ transport were evaluated the inhibition was also concentration-dependent but DMC had a more potent effect. Thus, 50 µM DMC or CLX caused inhibition of 97 or 85%, respectively (Figure 2B). The Ca^2+^/P_i_ coupling deduced from the initial rates of Ca^2+^ transport and ATP hydrolysis was close to 2 in the absence of drug but decreased as the drug concentration was raised. The coupling ratio was lower in the presence of DMC than in the presence of CLX for a given drug concentration (Figure 2B, inset).

**Figure 3:**
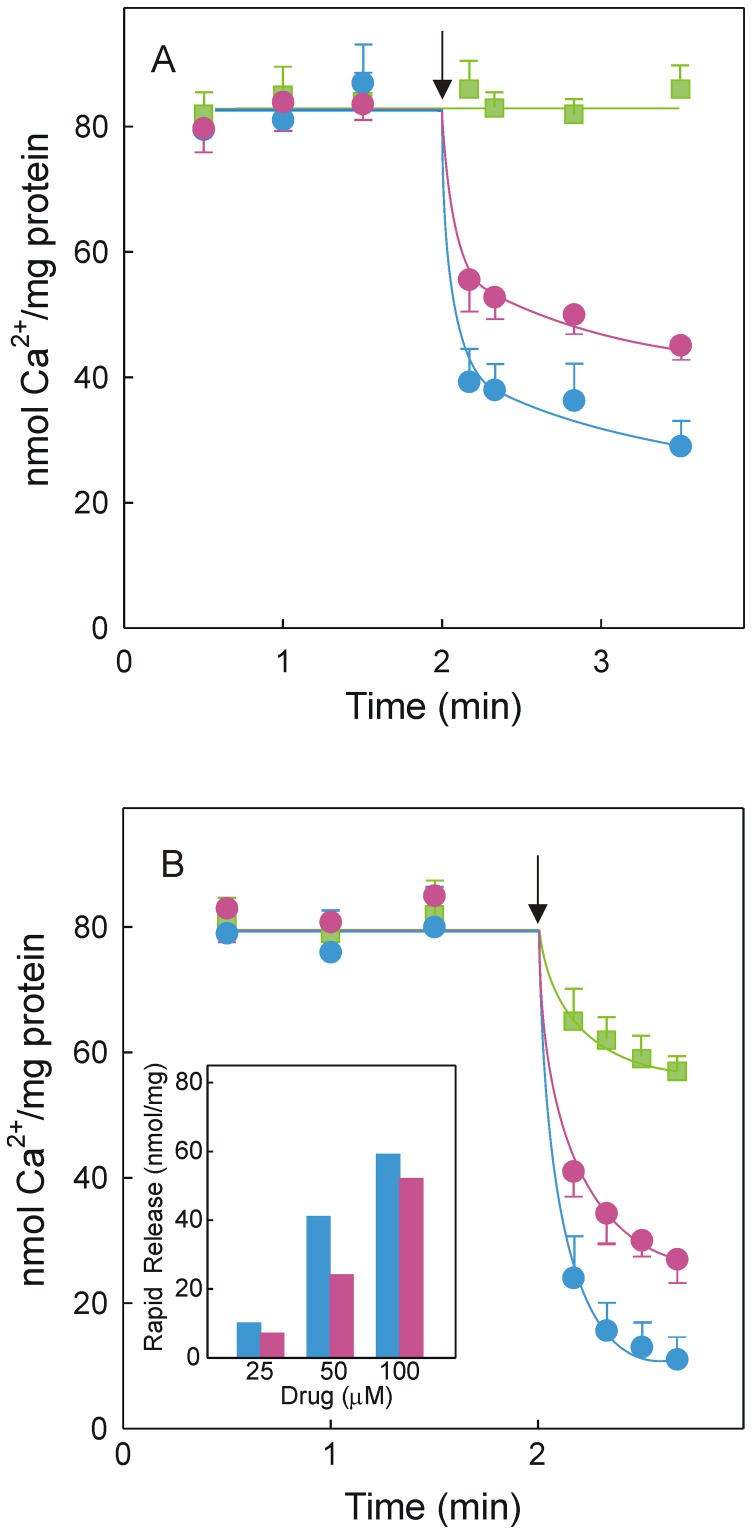
Drug effect on Ca^2+^-loaded vesicles. The Ca^2+^ loading medium was 20 mM Mops, pH 7.0, 80 mM KCl, 5 mM MgCl_2_, 0.2 mM EGTA, 0.138 mM [^45^Ca]CaCl_2_, 0.05 mg/ml native SR vesicles and 1 mM ATP. Reaction medium aliquots were filtered at different times and processed to evaluate ^45^Ca^2+^ retained by the vesicles. (A) Samples were left untreated (green squares) or supplemented with 50 µM DMC (blue circles) or 50 µM CLX (red circles) after 2 min of reaction. (B) The addition after 2 min was 5 mM EGTA (green squares), 5 mM EGTA plus 50 µM DMC (blue circles) or 5 mM EGTA plus 50 µM CLX (red circles). Times of addition are marked by an arrow. Inset, rapid component of Ca^2+^ release in the presence of DMC (blue bars) or CLX (red bars) when the Ca^2+^-pump was arrested. Data in the inset correspond to the first time point after drug addition once the EGTA-induced component was subtracted.

The above described results prompted us to examine the uncoupling mechanism. In these experiments, native SR vesicles were actively loaded with ^45^Ca^2+^ and then left untreated or supplemented with 50 µM drug. Drug addition decreased the pre-formed Ca^2+^ gradient and the extent of rapid Ca^2+^ release was higher with DMC than with CLX (Figure 3A). Alternatively, SR vesicles actively loaded with ^45^Ca^2+^ were supplemented with 5 mM EGTA to stop the functioning of the Ca^2+^-pump. Under these conditions, a steep Ca^2+^ gradient was created and a slow and passive Ca^2+^ release was developed (Figure 3B). When the experiment was repeated but EGTA addition was accompanied by 50 µM drug, the rapid component of Ca^2+^ release was still present. Using different drug concentrations it was demonstrated that membrane permeability increased as the drug concentration increased when the Ca^2+^-pump was arrested (Figure 3B, inset). The effect was greater for DMC than for CLX, whether or not the Ca^2+^-pump was functioning.

### Partial reactions

**Figure 4:**
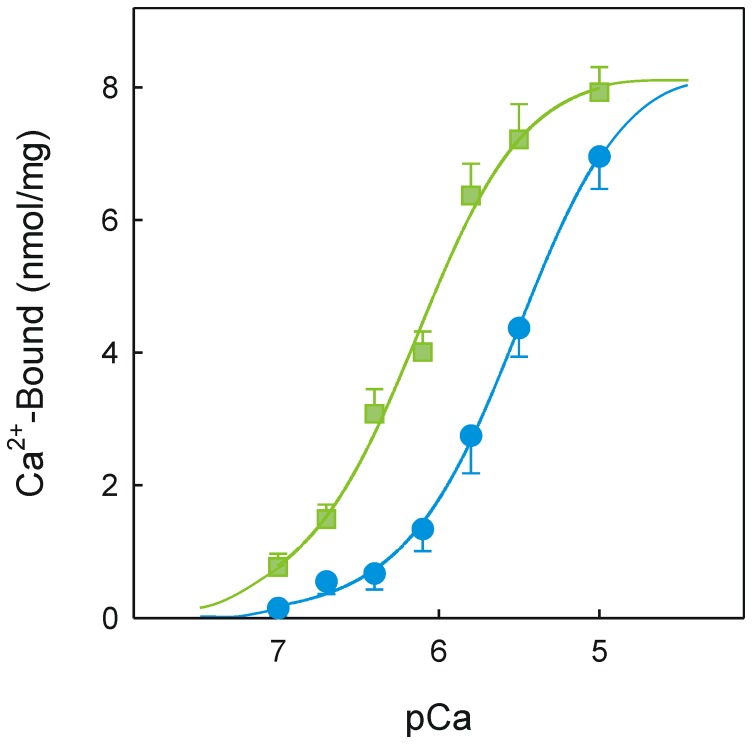
Effect of DMC on Ca^2+^ binding to the transport sites. The incubation medium consisted of 20_2_, 0.1 mM EGTA, 1 mM glucose, 0.2 mg/ml native SR vesicles in the absence (green squares) or presence (blue circles) of 50 µM DMC. Aliquots of 0.6 ml were mixed with a certain volume of medium containing 20 mM Mops, pH 7.0, 80 mM KCl, 3 mM MgCl_2_, 0.1 mM EGTA, 3 mM ^45^CaCl_2_ and 1 mM [^3^H]glucose. Samples were processed to determine specific ^45^Ca^2+^ bound.

The transport mechanism of the SR Ca^2+^-pump involves a sequence of conformational transitions whereby the translocation of two Ca^2+^ ions inside the sarco-endoplasmic reticulum is coupled to the hydrolysis of one ATP. Once the inhibition effect of DMC had been confirmed, a deeper analysis was undertaken by studying partial reactions of the catalytic cycle [36]. When Ca^2+^ binding to the high affinity transport sites was evaluated, a maximum concentration of ∼8 nmol Ca^2+^/mg protein and positive binding cooperativity were observed (Figure 4) as previously seen [37]. Moreover, when the Ca^2+^ binding titration was performed but the vesicles were exposed to 50 µM DMC before the addition of ^45^Ca^2+^, the apparent dissociation constant increased from 0.66 to 2.74 µM, whereas the maximum binding capacity and binding cooperativity remained unaltered. These data indicate that DMC stabilizes the inactive Ca^2+^-free conformation of the pump.

**Figure 5:**
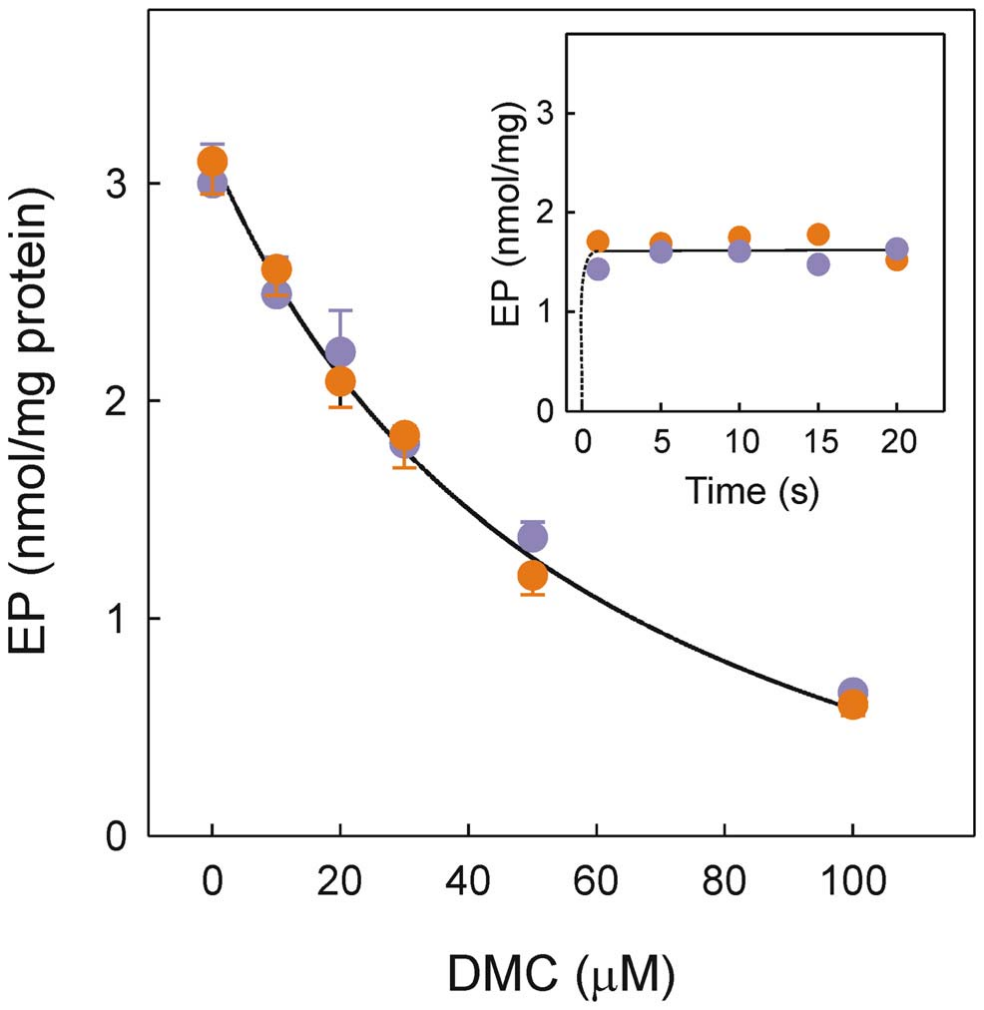
Effect of DMC on the steady-state EP level. The experiments were performed at ice-water temperature. The final reaction mixture was 20 mM Mops, pH 7.0, 80 mM KCl, 5 mM MgCl_2_, 1 mM EGTA, 0.686 mM CaCl_2_, 0.2 mg/ml SR vesicles, 8 µM A23187, 40 µM [γ-^32^P]ATP and a given DMC concentration when indicated. SR vesicles in the presence of Ca^2+^ were exposed to DMC and then mixed with radioactive ATP (orange circles) or SR vesicles in the absence of Ca^2+^ were exposed to DMC and then mixed with Ca^2+^ plus radioactive ATP (purple circles). The reaction was acid quenched after 5 s. Inset, time course of EP accumulation when the vesicles were exposed to 30 µM DMC in the presence (orange circles) or absence (purple circles) of Ca^2+^ before phosphorylation.

The EP accumulated during the functional activity of the pump was then studied at ice-water temperature. Phosphorylation of leaky vesicles by radioactive ATP established a steady-state level of ∼3.1 nmol EP/mg protein (Figure 5). However, when the vesicles in a Ca^2+^–containing medium were equilibrated with 30 µM DMC before phosphorylation, EP decreased to 1.7 nmol/mg protein (Figure 5, inset). The result was exactly the same when the vesicles in a Ca^2+^–free medium were equilibrated with 30 µM DMC before the addition of Ca^2+^ and radioactive ATP. When the experiments were extended to other DMC concentrations it was patent that EP decreased as the DMC concentration was raised from 10 to 100 µM (Figure 5). Data plotted in the main panel correspond to a phosphorylation time of 5 s. It is noteworthy that the DMC effect on the kinetics of EP accumulation that was observed in less than 1 s (Figure 5, inset) and the dependence of EP on DMC concentration (Figure 5) was independent of whether Ca^2+^ or DMC was added first.

**Figure 6:**
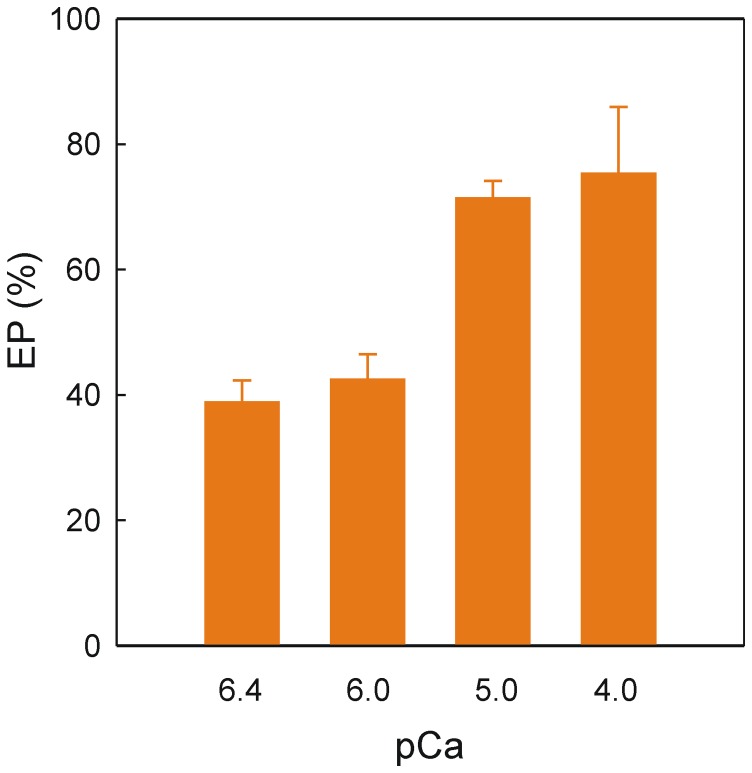
EP levels in the presence of DMC at various Ca^2+^ concentrations. The final reaction medium at ice-water temperature was 20 mM Mops, pH 7.0, 80 mM KCl, 5 mM MgCl_2_, 1 mM EGTA, CaCl_2_ to give a certain pCa, 0.2 mg/ml SR vesicles, 8 µM A23187, in absence or presence of 50 µM DMC and 40 µM [γ-^32^P]ATP. Phosphorylation was initiated by adding radioactive ATP to SR vesicles in the presence of Ca^2+^ and stopped 5 s later by acid quenching. The EP level at each pCa in the absence of DMC gaves the corresponding 100% value in the ordinate axis.

The potential effect of Ca^2+^ on EP was then estimated by equilibrating leaky vesicles in the presence of different Ca^2+^ concentrations with 50 µM DMC before the 5 s phosphorylation by adding [γ-^32^P]ATP. At low Ca^2+^ concentrations, equivalent to pCa 6.4 or 6.0, EP reached values of ∼40% with respect to control EP measured in the absence of drug (Figure 6). However, when the Ca^2+^ concentration was raised to give pCa values of 5.0 or 4.0 the EP level increased to ∼73%.

**Figure 7:**
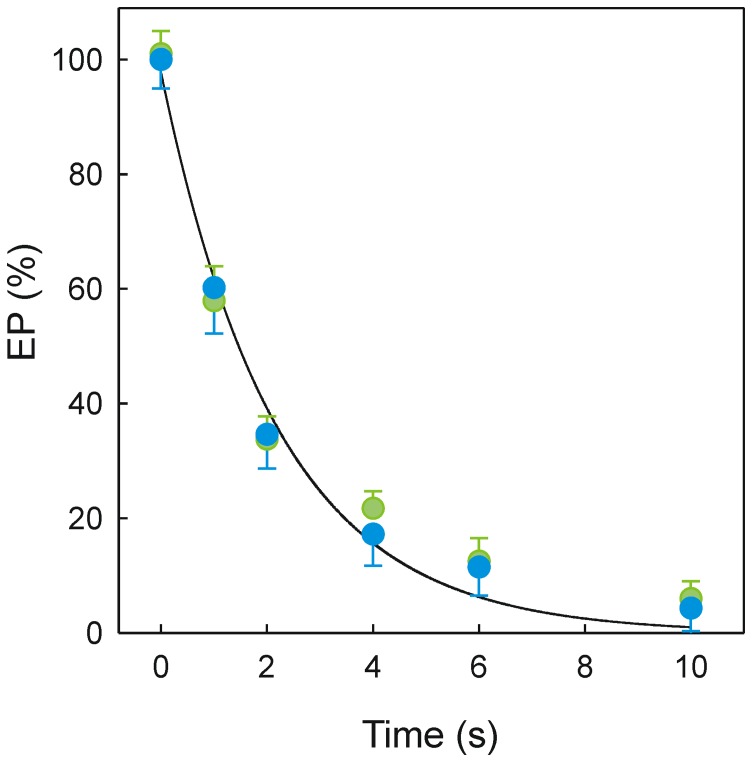
Kinetics of EP decay under turnover conditions. The experiments were performed at ice-water temperature. The phosphorylation medium was 20 mM Mops, pH 7.0, 80 mM KCl, 5 mM MgCl_2_, 1 mM EGTA, 0.686 mM CaCl_2_, 0.4 mg/ml SR vesicles, 16 µM A23187 and 40 µM [γ-^32^P]ATP in the absence (green circles) or presence (blue circles) of 50 µM DMC. After 5 s, the reaction mixture was 10-fold diluted with a medium containing non-radioactive ATP plus Ca^2+^ and EP was quenched by acid at different times.

EP decomposition is a partial reaction that can be resolved at low temperature by a pulse and chase experiment. In this case, leaky vesicles in a medium containing Ca^2+^ were left untreated or equilibrated with 50 µM DMC and then phosphorylated by 40 µM [γ-^32^P]ATP. After 5 s, the reaction mixture was 10-fold diluted with a medium containing 40 µM ATP to maintain the catalytic turnover. When the kinetics of [^32^P]EP decomposition was evaluated at different times a mono-exponential decay was observed that was not affected by the presence of DMC (Figure 7).

### Ligand-protein interaction

**Figure 8:**
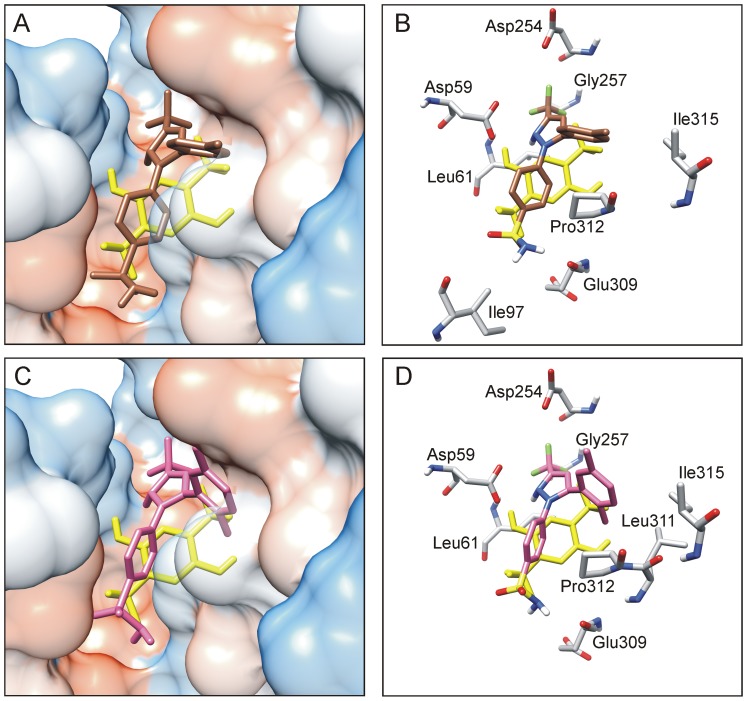
Computational docking of CLX and DMC compared to BHQ in the SERCA protein. Electrostatic potential surface of the BHQ binding cavity with CLX (A) or DMC (C) bound. Protein residues polarity, from non-polar to polar, is shown in a scale from red to white and blue. CLX (brown) and DMC (magenta) are shown in stick representation. BHQ (yellow) is included as a reference. Detail of SERCA residues at the BHQ site involved in interaction with CLX (B) or DMC (D). Interacting residues are denoted by grey sticks and protein structure was removed for the sake of clarity. Docking-predicted poses correspond to binding conformations with the lowest free energy change.

Information on the binding pocket of CLX and DMC in the SERCA protein was then addressed. The use of docking methodology generated several binding poses for each molecule that were ranked according to binding free energy scores. One conformation for each ligand with the most negative free energy change was selected and showed to fit in the binding pocket for the BHQ inhibitor (Figure 8). Simulation results predicted participation of residues Asp59, Leu61, Val62, Leu65, Pro308, Leu311 and Pro312. Likewise, CLX binding suggested participation of Asp59, Leu61, Ile97, Asp254, Gly257, Glu309, Pro312 and Ile315 whereas DMC binding involved Asp59, Leu61, Asp254, Gly257, Glu309, Leu311, Pro312 and Ile315. The thermodynamic K_d_ for CLX and DMC derived from the Gibbs equation after docking simulation was around 0.6 µM.

## Discussion

Mammalian cells express several SERCA isoforms with a characteristic tissue distribution being considered SERCA2b the housekeeping isoform due to its ubiquitous presence [38]. Although they possess distinct carboxyl terminal sequences and differ in Ca^2+^ binding affinity and maximal turnover rate, all of them share the same functional mechanism.

Our *in vitro* assays with leaky vesicles demonstrated that DMC is an inhibitor of the SR Ca^2+^-pump, showing inhibition potency somewhat lower than CLX with respect to Ca^2+^-ATPase activity (Figure 1). Inhibition by CLX was previously reported using a microsomal preparation of PC-3 cells [16]. Data obtained with native vesicles revealed that CLX was a more potent inhibitor of Ca^2+^–ATPase activity than DMC (Figure 2A) but DMC was a more potent inhibitor of the ATP-dependent Ca^2+^ transport than CLX (Figure 2B). In any case, Ca^2+^ transport was more sensitive to inhibition than Ca^2+^-ATPase activity and therefore the drugs under study induced Ca^2+^/P_i_ uncoupling in a concentration-dependent manner (Figure 2B, inset).

Uncoupling due to increased membrane permeability will produce rapid Ca^2+^ release from SR vesicles and rapid release of Ca^2+^ from Ca^2+^-loaded vesicles was observed when DMC or CLX was added (Figure 3). Rapid Ca^2+^ release occurred when the Ca^2+^-pump was partially operative (Figure 3A), or arrested by excess EGTA (Figure 3B), indicating that the uncoupling effect was not related with the functioning of the pump. Furthermore, the rapid Ca^2+^ release was dependent on drug concentration and was greater in the presence of DMC (Figure 3B, inset). The concentration dependence observed cannot be reconciled with a totally unspecific solubilization/leak process mediated by membrane phospholipids. Rapid increase of cytosolic Ca^2+^ was previously observed when DMC or CLX but not other COX inhibitors were added to U251 tumor cells [17]. Permeabilization activity induced by CLX was also detected in unillamelar [39] but not in multilamellar liposomes [16]. Assays with microsomal preparations have identified an alternative mechanism of uncoupling so-called “slippage of the pump” that occurs when P_i_ is released from the ternary complex E⋅P⋅Ca_2_ before Ca^2+^ is released inside the SR [40], [41]. EP-mediated intramolecular uncoupling produces Ca^2+^ dissociation into the external (cytosolic) space that prevents Ca^2+^ transport but does not induce Ca^2+^ release, so that this type of uncoupling can be ruled out.

Ca^2+^-free and Ca^2+^-bound conformations that may or not be phosphorylated are key molecular structures involved in the pumping mechanism. Ca^2+^ binding to the pump on the cytosolic side triggers a large structural rearrangement [42] that is necessary for autophosphorylation. Direct measurements of Ca^2+^ binding (Figure 4) indicated that DMC stabilizes the inactive Ca^2+^-free conformation therefore DMC interferes with the Ca^2+^ binding process.

The reaction of Ca^2+^–bound conformation with MgATP leads to rapid accumulation of the intermediate species EP. It is apparent that EP decreased when the DMC concentration was raised (Figure 5) reproducing the dependence observed for inhibition of the Ca^2+^-ATPase activity (Figure 1). These *in vitro* effects of DMC took place in the same concentration range as CLX elicited responses unrelated with COX-2 inhibition [43]. The dependence of EP on DMC can be explained by retention of the inactive Ca^2+^–free conformation that is dependent on drug concentration.

Certain pump inhibitors, including TG, exert the effect when exposed to the Ca^2+^-free but not to the Ca^2+^-bound conformation [44]–[46]. As a consequence, the kinetics of EP accumulation in the presence of inhibitor is developed in the second time frame when measured at low temperature and is dependent on the pump conformation exposed to the inhibitor [44]–[46]. In the present case, no difference was found when the Ca^2+^-free conformation (i.e., SR vesicles in the absence of Ca^2+^) or Ca^2+^-bound conformation (i.e., SR vesicles in the presence of Ca^2+^) were exposed to the drug before phosphorylation (Figure 5). This indicated no preferential interaction of DMC with the Ca^2+^-free conformation. In other words, the Ca^2+^-bound conformation was not protected from DMC and therefore DMC rapidly shifted the Ca^2+^ binding equilibrium retaining the Ca^2+^-free conformation. Moreover, the mass effect showing partial recovery of the EP level in the presence of DMC and high Ca^2+^ (Figure 6) was consistent with the mentioned effect of DMC on Ca^2+^ binding. These data suggest that the population of transporter with Ca^2+^-bound conformation during the catalytic turnover in the presence of DMC increases when Ca^2+^ is raised but does not reach the EP level observed in the absence of inhibitor.

Conformational transitions of EP species lead to Ca^2+^ translocation across the membrane and then hydrolysis of EP to complete the reaction cycle. The kinetics of EP decomposition that is considered a rate-limiting step was not affected by the presence of a DMC concentration producing 60% inhibition of the Ca^2+^-ATPase activity (Figure 7).

BHQ is a transmembrane inhibitor of the SR Ca^2+^-pump that stabilizes the Ca^2+^-free conformation. The stabilization involves hydrogen bonds between the two hydroxyl groups of BHQ, one with Asp59 on transmembrane helix M1 and the other with Pro308 on transmembrane helix M4 [47]. The BHQ binding pocket appears to be quite tolerant since a large number of bulky structures containing up to three aromatic rings tethered to each other have been characterized as novel SERCA inhibitors by computational docking [48], [49]. Large inhibitors protrude from the binding pocket and many of them present a characteristic 90^o^ bend.

DMC is a 1,5-diaryl-substituted pyrazole containing dimethylphenyl and benzenesulfonamide moieties. Molecular modeling applied to CLX and related structures indicated that the terminal phenyl ring and the polar carboxamide or sulfonamide group at the other extreme of the molecules appear approximately perpendicular to each other in the 3D structure [50]. Ligand docking simulation for BHQ perfectly fitted the crystallographic information available (Figure 8). Our data also predict hydrogen bonding between the polar sulfonamide group of CLX or DMC and Glu309 inside the pocket as well as location of the hydrophobic phenyl ring at 90^o^ in the site entrance. The docking pose also reveals altered ligand orientation when the phenyl ring substituent 4-methyl was changed to 2,5-dimethyl, i.e., CLX *vs*. DMC. This is consistent with the observed difference in the SERCA residues that are involved in the CLX or DMC-protein interaction.

In conclusion, DMC binding occurs at the BHQ binding site and the effect is a combination of Ca^2+^-pump inhibition by interfering with Ca^2+^ binding and uncoupling related with membrane permeabilization activity. Both actions can explain the cytotoxic and anti-proliferative effects that have been attributed to the drug. It remains to be established how *in vivo* selectivity of DMC on tumor cells is achieved.
